# Towards a generalized vision of oxides: disclosing the role of cations and anions in determining unit-cell dimensions

**DOI:** 10.1107/S0108768110013200

**Published:** 2010-05-06

**Authors:** Ángel Vegas, Maurizio Mattesini

**Affiliations:** a Instituto de Química Física ‘Rocasolano’, CSIC, C/Serrano 119, Madrid E-28006, Spain; b Departamento de Física de la Tierra, Astronomía y Astrofísica I, Universidad Complutense de Madrid, Madrid E-28040, Spain

**Keywords:** electron localization, high-pressure experiments

## Abstract

Theoretical calculations of the electron-localization function show that, at the volumes of the two CaO phases (rocksalt and CsCl type), the parent Ca structures (*fcc*: face-centred cubic and *sc*: simple cubic, respectively) exhibit charge concentration zones which coincide with the positions occupied by the O atoms in their oxides. Similar features, also observed for the pairs Ca/CaF_2_ and BaSn/BaSnO_3_, are supported by recent high-pressure experiments as well as electron-localization function (ELF) calculations, carried out on elemental K. At very high pressures, the elemental K adopts the *h*P4 structure, topologically identical to that of the K atoms in high-pressure K_2_S and high-temperature α-K_2_SO_4_. Moreover, the ELF for the *h*P4 structure shows charge concentration (∼ 2 electrons) at the sites occupied by the S atoms in the high-pressure K_2_S phase. All these features confirm the oxidation/high-pressure equivalence as well as the prediction of how cation arrays should be metastable phases of the parent metals. For the first time to our knowledge, the structure type, dimension and topology of several oxides and fluorides (CaO, CaF_2_ and BaSnO_3_) are explained in univocal physical terms.

## Abstract 2

Theoretical calculations of the electron-localization function show that, at the volumes of the two CaO phases (rocksalt and CsCl type), the parent Ca structures (*fcc*: face-centred cubic; *sc*: simple cubic) exhibit charge-concentration zones which coincide with the positions occupied by the O atoms in their oxides. For the first time, the structure type, dimension and topology of CaO and BaSnO_3_ are explained in univocal physical terms.

## 1. Introduction

The approach of Pauling (1960 ▶; small cations lodged in the voids of close-packed arrays of bulky anions) has been the paradigm for describing crystal structures during the last century. Although commonly accepted, it has not offered a satisfactory explanation of crystal structures, most of which remain misunderstood.

A critical survey of the limitations of the ionic model can be found in the works of O’Keeffe & Hyde (1981 ▶, 1985 ▶), where alternative approaches based on the cation substructures have been suggested. One of the challenges originating from these new insights is that, in many instances, the structure of a given alloy is preserved in its corresponding oxide (Vegas, 2000 ▶; Vegas & Jansen, 2002 ▶). Two singular examples are the pairs BaSn/BaSnO_3_ and *sc*-Ca/CaO (CsCl type), in which the dimensions of the BaSn (CsCl type) and of simple cubic Ca (*sc*-Ca) cells are maintained in the respective oxides (Beck & Lederer, 1993 ▶; Martínez-Cruz *et al.*, 1994 ▶; Vegas & Tranqui, 1996 ▶; Vegas, 2000 ▶).

The challenge arises, in part, from the contrast between an ionic model which sees the oxides as a network of isolated ions, on one hand, and the simplest model for metallic crystals proposed by Thomas (1927 ▶), Fermi (1927 ▶) and Dirac (1930 ▶), in which the valence electrons are smeared out as an electron sea, generating a quasi-planar surface of charge density. However, recent analysis of the topology of the electron density reveals the existence of inhomogeneities, such as those found in the *bcc* phases of the alkali metals by Luaña *et al.* (2003 ▶).

The results derived from these types of calculations are connected with older models that contemplate the bonding in metals and alloys, like electrides. The term electride is used to mean that valence electrons of a metal are not delocalized but concentrated at the voids of the networks, acting as pseudo-anions. This old idea revived by Nesper (1991 ▶), citing an idea of von Schnering, has been supported by numerous empirical facts and by quantum-mechanical formalisms of the electron-localization function (ELF; Savin *et al.*, 1997 ▶).

The idea resulting from these studies is simple and has been clearly defined as the AIMM model (anions in metallic matrices model) by Vegas *et al.* (2006 ▶). According to this model, the crystal structure of many inorganic compounds can be understood as a metallic matrix acting as a host lattice for non-metallic atoms, the electron density of the metal inducing the final positions of the non-metallic atoms in the crystal. It should be remarked that in this scenario, the concept of the metallic matrix is not only restricted to a dense packing of atoms, as in metals and/or alloys, but also applies to more open skeletons. Examples are the structures of the Al*X*_3_ family (*X* = F, Cl, OH; Vegas *et al.*, 2006 ▶) and the skeletons of aluminates (Santamaría-Pérez & Vegas, 2003 ▶) and silicates (Santamaría-Pérez *et al.*, 2005 ▶). This idea also finds support in the theoretical calculations reported for PB (zincblende-type) in which, at elevated pressures, the electron pairs of the P—B bonds separate to form non-nuclear maxima (NNM) in between the two atoms (Mori-Sánchez *et al.*, 2001 ▶).

More recently, high-pressure experiments on Na and K have increased interest in this problem. Thus, two new phases (*o*P8 and *h*P4) have been reported for Na and K. In the case of Na, the *o*P8 phase (*Pnma*), isolated at 117 GPa by Gregoryanz *et al.* (2008 ▶), transforms at 200 GPa in the hexagonal *h*P4 phase (*P*6_3_/*mmc*; Ma *et al.*, 2009 ▶). The same structures were observed for K by Lundegaard *et al.* (2009 ▶) and by Marqués *et al.* (2009 ▶) at *ca* 20 and 50 GPa. Interestingly, the work of Marqués *et al.* (2009 ▶) on *h*P4-K includes calculations of the ELF at 25 GPa, showing maxima located at the (2*d*) sites (1/3, 2/3, 1/4), of the space group * P*6_3_/*mmc*. These basins contained around 2 electrons and were interpreted as Lewis pairs (LPs) acting as pseudoanions. Note that these sites are where the S atoms are located in the high-pressure phase of Na_2_S (also *P*6_3_/*mmc*; Vegas *et al.*, 2001 ▶), as noted by Marqués *et al.* (2009 ▶).

These results confirm that, at elevated pressures, the valence electrons can form LPs converting the metals (in this case *h*P4-K) into true ionic structures. This revival of the concept of metals as electrides confirms the validity of the AIMM model (Vegas *et al.*, 2006 ▶), and has led us to extend it to other similar compounds. An instructive example is CaO, which undergoes the B1 (NaCl) → B2 (CsCl) transition at 60–70 GPa (Jeanloz *et al.*, 1979 ▶). The interest behind this compound resides in the fact that the unit cell of the B2 phase (2.64 Å) is surprisingly close to the unit cell of *sc*-Ca (2.62 Å). Thus, under pressure the Ca atoms adopt the same structure and cell dimensions in both element and oxide. Such an enigmatic behaviour was also noticed in other structure pairs such as Ca_2_Si/β-Ca_2_SiO_4_ (O’Keeffe & Hyde, 1985 ▶), BaSn/BaSnO_3_ (Martínez-Cruz *et al.*, 1994 ▶), LnAl/LnAlO_3_ (Ramos-Gallardo & Vegas, 1997 ▶) and Cs_3_Bi/Cs_3_BiO_3_ (Zoche & Jansen, 1997 ▶), but has remained unexplained to date.

For this reason we have undertaken the present study based on the calculation of the ELFs for the *fcc*, *bcc* and *sc* phases of Ca (Olyjnik & Holzapfel, 1984 ▶), to see whether electron-localized function plots could help us to understand the behaviour of the related oxide structures. The study was further extended to the pairs Ca/CaF_2_ and BaSn/BaSnO_3_.

## 2. Structures of Ca and CaO

**Figure 1:**
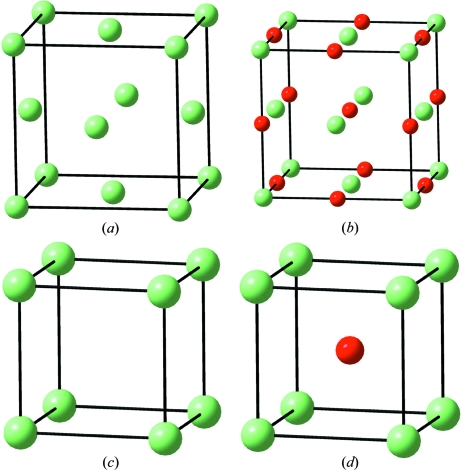
(*a*) The unit cell of *fcc*-Ca under ambient conditions. (*b*) The unit cell of the NaCl-type structure of CaO under ambient conditions: O (red) and Ca atoms (light green). (*c*) The simple cubic phase of Ca (α-Po structure) obtained at 32 GPa. (*d*) The CsCl-type structure of CaO obtained at 60–70 GPa. Colours as in (*b*).

The phase transitions and the structures of both Ca and CaO are represented in Fig. 1 ▶. Elemental Ca is *fcc* (*a* = 5.58 Å) at ambient conditions. At 723 K it transforms to *bcc* (*a* = 4.38 Å). However, under pressure it undergoes the transition sequence *fcc* → *bcc* (body-centred cubic) → *sc* (single cubic, α-Po structure) at 19.5 and 32 GPa (Olyjnik & Holzapfel, 1984 ▶). The unit-cell parameters of these high-pressure phases are *a* = 3.56 and 2.62 Å. Olyjnik & Holzapfel (1984 ▶) found that the two *bcc* phases, observed at 723 K and at 19.5 GPa, are identical and can be correlated by a common equation-of-state (EOS).

Regarding the oxide, CaO is rocksalt at ambient conditions, undergoing the B1 (NaCl) → B2 (CsCl) transition at 60–70 GPa (Jeanloz *et al.*, 1979 ▶). Their unit-cell dimensions are *a* = 4.80 (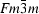) and 2.64 Å (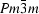). These structures are collected in Fig. 1 ▶.

By comparing Figs. 1 ▶(*a*) and (*b*) it can be seen that the *fcc* structure of Ca is maintained in the rocksalt-like structure of CaO, although oxidation produces a significant contraction from 5.58 to 4.80 Å. In previous work (Vegas, 2000 ▶), this contraction was attributed to the pressure exerted by the O atoms as it occurs in many other oxides (Vegas, 2000 ▶; Vegas & Jansen, 2002 ▶). Nonetheless, these are qualitative considerations based on the equivalence oxidation/pressure, and therefore give a far from satisfactory explanation of this large contraction (∼ 63% by volume).

However, it is worth noting here that the unit-cell parameter of the rocksalt-like CaO is within the range of stability of the *fcc* phase of calcium (Vegas, 2000 ▶). Thus, making use of the EOS, the unit-cell volume of *fcc*-Ca at the transition is 102.62 Å^3^ (*a* = 4.68 Å), which is slightly below the value of 4.80 Å for CaO. In other words, if we could eliminate the O atoms in the rocksalt CaO, the remaining Ca subarray would correspond to the structure of *fcc*-Ca at a pressure slightly lower than that of the transition to the *bcc* phase (19.5 GPa). This could explain, at least at a qualitative level, why CaO preserves the *fcc* substructure of Ca.

**Figure 2:**
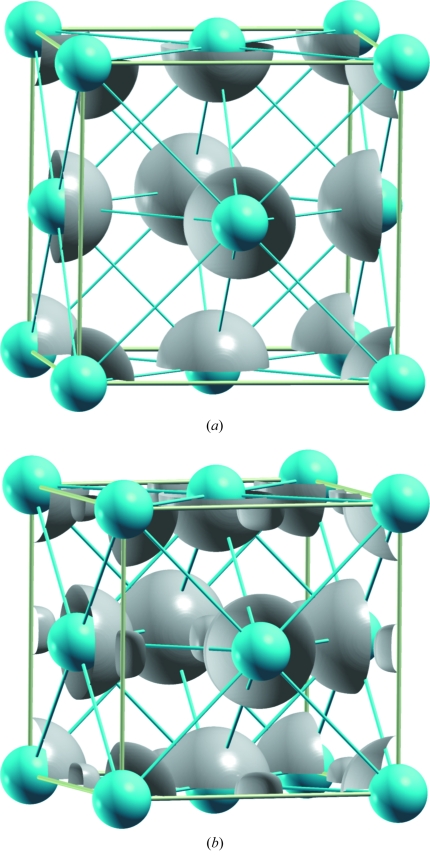
ELF for the *fcc* structure of Ca. (*a*) Unit cell at ambient conditions (*a* = 5.521 Å) showing the charge concentration at the atomic sites. (*b*) Computed ELF for an *fcc*-Ca unit cell having the same dimension as the rocksalt CaO phase (*a* = 4.829 Å). Note the formation of a charge concentration at the oxygen positions. The three-dimensional ELF plots have been generated with the help of the XcrySDen molecular structure visualization program (Kokalj, 2003 ▶), using an isovalue of 0.8 and applying the tricubic spline interpolation with a degree of three. Blue spheres represent Ca atoms.

Now if we compare the structure of *sc*-Ca with that of CaO (B2; see Figs. 2 ▶ *c* and *d*) we notice that, as for the B1 phase, the *sc*-Ca structure is also preserved in the B2 phase of the oxide. Here, the similarity is more striking because the dimensions of the respective cells are almost identical.

The underlined Ca—CaO structural singularities lead to two important questions that remain unanswered so far:

(i) Why do the *sc*-Ca and the B2 phase of CaO have identical networks and dimensions?
(ii) Why does *fcc*-Ca reduce its volume by precisely 63% when the B1 phase of CaO is being formed?

Therefore, the crucial issue is not only why a given compound adopts a certain structure, but also whether we are able to explain their dimensions by physical arguments. Here we try to address the above matters by applying the calculated ELFs for the three Ca phases (*fcc*, *bcc* and *sc*).

## 3. Method used to perform the calculations

The density-functional theory (DFT) calculations (Hohenberg & Kohn, 1964 ▶; Kohn & Sham, 1965 ▶) were carried out for *sc*-, *fcc*- and *bcc*-Ca using the Perdew–Burke–Ernzerhof (PBE; Perdew *et al.*, 1996 ▶) approximation for the exchange-correlation function, and the pseudo-potential plane-wave method within the *quantum-ESPRESSO* software package (Giannozzi *et al.*, 2009 ▶). Specifically, the core-valence interactions have been described through the Vanderbilt ultrasoft pseudo­potentials (Vanderbilt, 1990 ▶), including nonlinear core correction and treating the Ca semicore *s* and *p* states as valence electrons (*i.e.* Ca [Ne] 3*s* ^2^3*p* ^6^4*s* ^2^). The electronic wavefunctions and the charge density have been expanded by using plane-wave basis sets defined by energy cutoffs of 70 and 700 Ry, respectively. The Brillouin-zone integration has been performed employing the Gaussian-broadening technique and using a converged reciprocal space **k**-point meshing. A smearing parameter of 0.002 Ry has been employed. The set of parameters used for these calculations allowed the computation of the equation-of-states for both *sc* and *fcc* phases that are in good agreement with the available experimental data (Yabuuchi *et al.*, 2005 ▶; Brandes & Brook, 1992 ▶). In addition, the *bcc*–*sc* transition pressure has been calculated to be around 39 GPa, a value that compares well with the experimental transition at 32 GPa (Olyjnik & Holzapfel, 1984 ▶) and *ab initio* calculations 40.4–41.2 GPa (Jona & Marcus, 2006 ▶).

Our theoretical investigation of the formation of Ca—O bonds has been carried out by a careful analysis of the bonding pattern through the ELF (Becke & Edgecombe, 1990 ▶). We recall here that the ELF is a scalar function of position that provides a measure of the probability of finding an electron in the neighbourhood of another electron with the same spin. Therefore, it quantifies the degree of electron localization with respect to the free-electron gas distribution. The dimensionless ELF magnitude ranges from 0.0 (no localization) to 1.0 (strong localization) with ELF = 0.5 corresponding to a perfect free-electron gas distribution. The latter case is most likely observed for metallic systems. We applied this criterion to obtain specific insights about possible topological relations between *sc*-, *fcc-* and *bcc*-Ca and their analogous oxides. Specifically, we start by imposing precise unit-cell volumes to the metallic networks and monitoring the evolution of the computed three-dimensional ELF pictures. This procedure provides the opportunity of detecting potential metastable oxides, as presented and commented on in the next section.

## 4. Discussion

We will begin our discussion by considering the second question posed above, that is why does CaO adopt the B1 structure and why should *fcc*-Ca shrink when O atoms are inserted. It should be emphasized here that such questions were (if ever) rarely formulated and therefore no clear explanations have been provided.

The ELF for *fcc*-Ca was firstly computed at theoretical ambient conditions (*a* = 5.521 Å). The result is shown in Fig. 2 ▶(*a*) where the basins appear located around the Ca atoms and where no NNM is visible. The charge is concentrated at the atomic positions, with the valence electron contribution probably smeared-out over the unit-cell volume.

However, when the volume of the unit cell of *fcc*-Ca is reduced to the theoretical value of the B1 structure of CaO (*a* = 4.829 Å), the ELF generates basins located at the same positions as the O atoms in CaO (see Fig. 2 ▶ *b*). This result clearly indicates that the precise dimensions of the B1 phase are those which force the smeared valence electrons of the Ca atoms to concentrate as LPs at (½, ½, ½). In other words, the unit cell of the B1 phase has the value at which formal anions (O^2−^) mimic the pressure at which the LPs are formed.

These results agree with those previously obtained for Ca_4_Sb_2_O (Savin *et al.*, 1997 ▶). This compound, initially taken as the Zintl phase Ca_4_Sb_2_ (Eisenmann & Schäfer, 1974 ▶; Hamon *et al.*, 1975 ▶), was later confirmed to be the suboxide Ca_4_Sb_2_O with the O atoms centring Ca_6_ octahedra (Eisenmann *et al.*, 1980 ▶). The calculation of the ELF on the O-free network Ca_4_Sb_2_ (Savin *et al.*, 1997 ▶) generated only one additional localization region at the position of O atoms in the experimentally observed Ca_4_Sb_2_O oxide. They also confirm our thoughts that O^2−^ anions would play the role of LPs, as advanced earlier for silicate skeletons (Santamaría-Pérez *et al.*, 2005 ▶) and also by *X* ^−^ anions in the aluminium halides (Vegas *et al.*, 2006 ▶).

**Figure 3:**
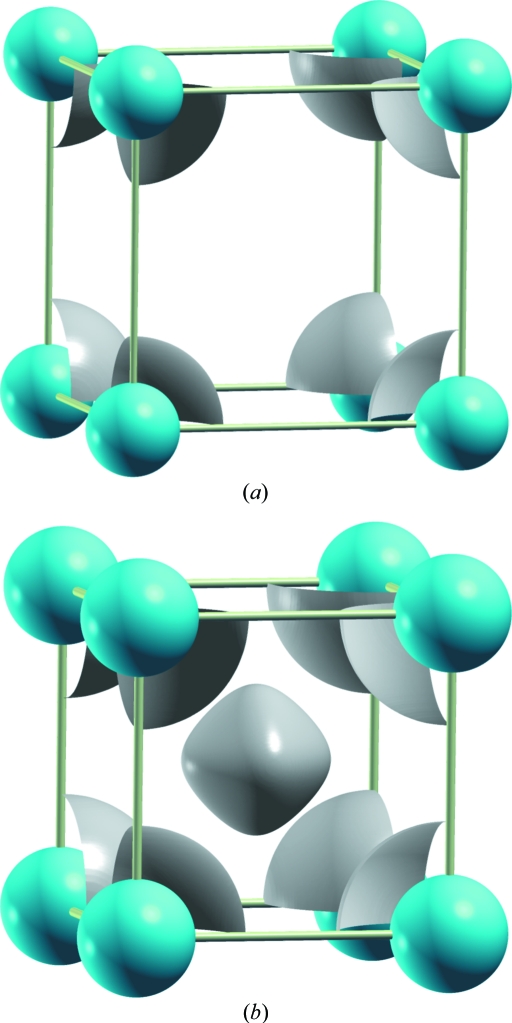
(*a*) ELF for the *sc*-Ca structure, with unit-cell parameter *a* = 3.498 Å (0 GPa). (*b*) ELF calculated with *a* = 2.645 Å (39 GPa), showing the charge concentration at the centre of the cell. Blue spheres represent Ca atoms.

The next step was to compute the ELF for the *sc*-Ca structure, stable in the pressure range 32–42 GPa (Olyjnik & Holzapfel, 1984 ▶). When the ELF is calculated at *a* = 2.645 Å, *i.e.* at the theoretical volume for the B2 phase of CaO, a localization region at the cell centre is again observed, coincident with the O atom in CaO (B2; Fig. 3 ▶ *b*). It should be remarked that the value of *a* = 2.645 Å is in very good agreement with the value of *a* = 2.615 Å measured for *sc*-Ca at 39 GPa (Olyjnik & Holzapfel, 1984 ▶). Again, the separation of the valence electrons to form an LP occurs at volumes identical to those of the corresponding oxide, giving additional support to the equivalence of LPs and anions. Interestingly, when the ELF is computed at *a* = 2.645 Å (39 GPa), the valence electrons remain attached to the atomic cores (*cf.* Figs. 3 ▶ *a* and *b*).

We have also mentioned the existence of a *bcc*-Ca. Unlike the B1 and B2 structures of CaO which reproduce the *fcc*- and *sc*-Ca structures, no binary nor ternary oxide of Ca with the *bcc*-Ca substructure has been reported so far. A candidate for such a compound could be a perovskite with the formula CaCa(OF_2_). Earlier attempts to synthesize this compound, by thermal decomposition of the mineral brenkite, Ca_2_(CO_3_)F_2_ (Leufer & Tillmanns, 1980 ▶), were unsuccessful, only leading to a mixture of CaF_2_ and CaO. This result was difficult to explain because if an ionic compound consists of isolated anions and cations, the formation of CaCa(OF_2_) should be possible.

However, when the ELF for the *bcc*-Ca is computed at several volumes, the failure in the synthesis becomes meaningful as there is no charge concentration at the voids of the metal structure. The fact that even at the volume of the *fcc* → *bcc* transition, occurring at 19 GPa (a = 3.56 Å), no charge concentration was observed, which could explain the non-existence of the oxyfluoride, probably as the F atoms will not find an isolated electron to satisfy its one-electron requirement.

**Figure 4:**
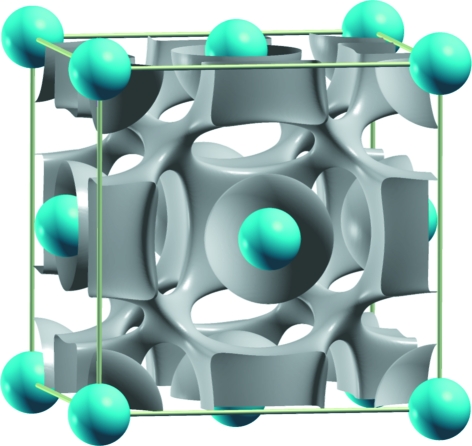
ELF computed for the *fcc*-Ca structure at a volume reduction of 6.3% (*a* = 5.408 Å), which is the volume of the fluorite CaF_2_ structure. This figure shows the presence of ELFs (isovalue of 0.5) at (¼, ¼, ¼), just at the centre of the tetrahedra occupied by the F atoms. Compare with the results obtained for CaO in Fig. 2 ▶(*b*). Blue spheres represent Ca atoms.

A final calculation was carried out on *fcc*-Ca with *a* = 5.408 Å. This parameter corresponds to the CaF_2_ structure, in which the *fcc* array of Ca is compressed only 6.3% in volume. As in CaF_2_ (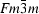) where the F atoms occupy the tetrahedral 8*c* sites (¼, ¼, ¼), it should be expected that the ELF would show charge concentration at these sites. As shown in Fig. 4 ▶, the ELF shows charge concentrations at the positions of the F atoms! Thus, the location of the F atoms is clearly coincident with this model and the fluorite structure can also be justified in terms of the charge distribution of the metal matrix for each volume (pressure).

## 5. BaSn and BaSnO_3_

At high pressure, BaSn undergoes the CrB → CsCl transition (Beck & Lederer, 1993 ▶). The isomorphism of both the perovskite BaSnO_3_ and the high-pressure phase of the BaSn (B2) (Martínez-Cruz *et al.*, 1994 ▶) suggests that an ELF calculation for B2 BaSn, should show localization at regions coincident with the oxygen positions in BaSnO_3_. A similar isomorphism was reported for the LnAl alloys and the perovskites LnAlO_3_ (Ramos-Gallardo & Vegas, 1997 ▶). This hypothesis is consistent with a Zintl phase approach to BaSn, when the transfer of Ba valence electrons converts Sn atoms into pseudo-Te atoms (Ψ-Te). Accordingly, the Sn-substructure is similar to the high pressure of Te (γ-Te), which is a rhombohedrally distorted simple cubic structure (*a* = 2.95 Å, α = 102.6°; Po-type). In this structure, the Te atoms have six nearest neighbours which should be bonded by two-centre, two-electron bonds. The O atoms, in BaSnO_3_, are located at the middle of these bonds. Recall the similarity of the γ-Te structure with that of TeO_3_ (FeF_3_- or AlF_3_-type) and how, at high temperature, the FeF_3_ (AlF_3_) structure transforms to a cubic ReO_3_-type structure.

**Figure 5:**
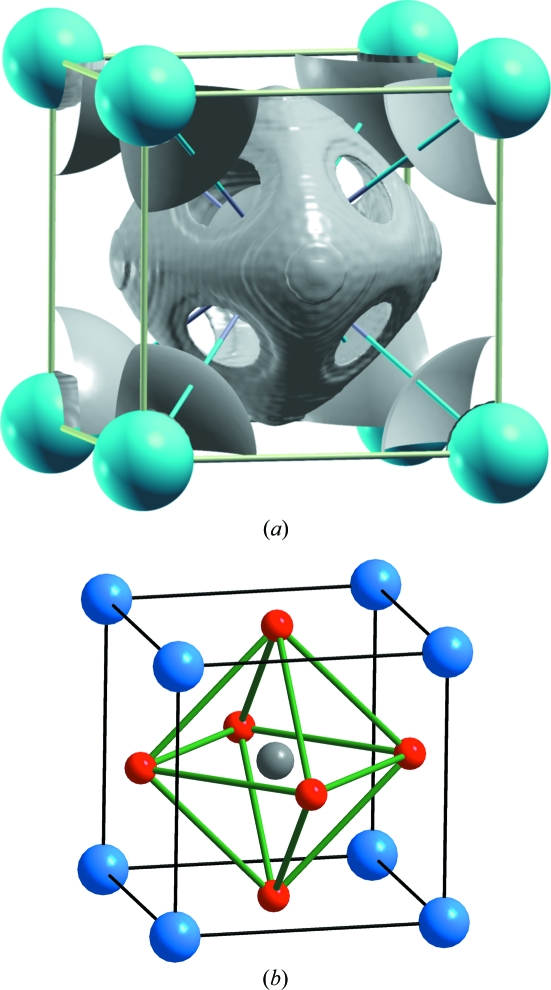
(*a*) ELF with an isovalue of 0.50 for the BaSn structure with a lattice constant of 4.070 Å. Note the formation of a free-electron density at the oxygen positions of the BaSnO_3_ perovskite phase. Blue spheres represent Ba atoms. The Sn atom is centring the cell and is hidden by the octahedral ELF. (*b*) The structure of BaSnO_3_, showing the B2 substructure of BaSn and the O octahedron around the Sn atom. Ba, Sn and O atoms are represented by blue, grey and red spheres, respectively.

In agreement with this description, the computation of the ELF shows a clear charge concentration that mimics the O_6_ octahedron surrounding each Sn atom. As previously seen in Fig. 5 ▶, the ELF presents maxima almost at the centre of the cube faces, exactly where the O atoms are located in the oxide (see Fig. 5 ▶ *b*).

## 6. Concluding remarks

The computation of the ELF for different phases of calcium (*fcc*, *bcc* and *sc*) discloses crucial aspects of these crystal structures which have been hidden for almost a century. Our results give strong support to the intuitive hypothesis that considered the metallic subnets in compounds as metastable structures of the parent metal. This hypothesis, formulated on the basis of the analogies existing between the cation arrays and high-pressure phases of the alloys, has been expressed as follows: ‘*If high pressure gives rise to a redistribution of the electrons and hence to a phase transition, similar results could be obtained if electrons are redistributed by the presence of* ***foreign atoms***’. This term was first coined to denote unlike atoms added to a parent metal structure when forming a compound. Later, it was seen that these ‘foreign atoms’ can act like pressure and/or temperature, provoking phase transitions in the parent metal structures (Vegas & Martínez-Cruz, 1995 ▶; Vegas, 2000 ▶).

**Figure 6:**
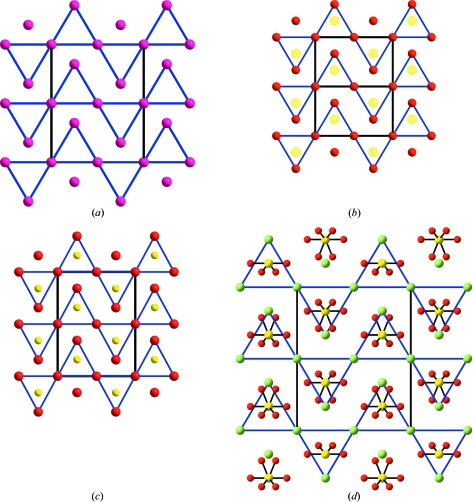
(*a*) The structure of *h*P4-K (*P*6_3_/*mmc*). (*b*) The *h*P4-K structure showing the charge concentration (yellow spots) obtained by the ELF calculations. (*c*) The orthorhombic (*Pmma*) structure of K_2_S at high pressure, showing its similarity with the *h*P4-K structure in (*b*). Its deviation from the ideal *P*6_3_/*mmc* of *h*P4-K is quite small, as seen from the unit-cell parameters *a* = 6.53, *b* = 5.09 (2), *c* = 8.77 Å, where the *c* axis is almost equal to 5.09 × (3)^1/2^ = 8.82 Å. (*d*) The high-temperature structure of K_2_SO_4_. The K atoms are represented by red circles in (*a*), (*b*) and (*c*), and by green circles in (*d*) where the red colour is used to denote the disordered O atoms. In all cases, the S atoms are represented by yellow circles.

This prediction becomes a categorical statement after the work of Marqués *et al.* (2009 ▶) where for the first time it is shown that, at very high pressures, the elemental K adopts the *h*P4 structure. This phase is topologically identical to that of the K atoms in the high-pressure K_2_S (Vegas & Jansen, 2002 ▶) and the high-temperature α-K_2_SO_4_. Moreover, the ELF for the *h*P4 structure shows charge concentration (about 2 electrons) at the sites occupied by the S atoms in K_2_S (*cf.* Fig. 6 ▶ *b* and *c*). The three structures, K, K_2_S and K_2_SO_4_, are drawn in Fig. 6 ▶.

These results led us to a second important conclusion, *i.e.* they provide strong physical meaning to the fact that the O atoms are located close to the positions of Lewis pairs as if they were there to ‘catch’ the electron pairs. These electrons can be either bonding pairs as in silicate networks (Santamaría-Pérez & Vegas, 2003 ▶; Santamaría-Pérez *et al.*, 2005 ▶) or isolated electrons occupying interstices of the metallic nets. As said by Vegas *et al.* (2006 ▶), the formal O^2−^ anions ‘*would play the same role as bonding pairs of electrons, whereas X^−^ anions would mimic a two-centre, one-electron bond*’.

A third consequence refers to the values of the unit cells in both *fcc*- and *sc*-Ca at which the ELF shows charge concentration. This feature can be stated in the following way: ‘*electrons are separated from the atomic cores (located) at pressures producing the same volume as that of the corresponding oxide. In other words, oxides try to reproduce the dimensions of the metal cell at which electrons are located at these sites*’. This postulate accounts for the dimensions of all the structures studied in this article. To our knowledge, this is the first time that not only the structure but also the dimensions of a compound can be explained unequivocally in physical terms.
